# Supplementary material for: Optimizing spectral quality with quantum dots to enhance crop yield in controlled environments
Source: Commun Biol. 2021 Jan 27;4:124. doi: 10.1038/s42003-020-01646-1 (PMC7840809; doi:10.1038/s42003-020-01646-1)
Supplement: Supplementary file 3 — Reporting Summary [file 42003_2020_1646_MOESM3_ESM.pdf]

nature research

Corresponding author(s): Matthew R. Bergren, Gene A. Giacomelli

Last updated by author(s): 12/05/2020

Reporting Summary

Nature Research wishes to improve the reproducibility of the work that we publish. This form provides structure for consistency and transparency in reporting. For further information on Nature Research policies, see our Editorial Policies and the Editorial Policy Checklist.

Statistics

For all statistical analyses, confirm that the following items are present in the figure legend, table legend, main text, or Methods section.

n/a	Confirmed	
☐	☒	The exact sample size (n) for each experimental group/condition, given as a discrete number and unit of measurement
☐	☒	A statement on whether measurements were taken from distinct samples or whether the same sample was measured repeatedly
☐	☒	The statistical test(s) used AND whether they are one- or two-sided<br>*Only common tests should be described solely by name; describe more complex techniques in the Methods section.*
☐	☒	A description of all covariates tested
☐	☒	A description of any assumptions or corrections, such as tests of normality and adjustment for multiple comparisons
☐	☒	A full description of the statistical parameters including central tendency (e.g. means) or other basic estimates (e.g. regression coefficient) AND variation (e.g. standard deviation) or associated estimates of uncertainty (e.g. confidence intervals)
☐	☒	For null hypothesis testing, the test statistic (e.g. F, t, r) with confidence intervals, effect sizes, degrees of freedom and P value noted<br>*Give P values as exact values whenever suitable.*
☒	☐	For Bayesian analysis, information on the choice of priors and Markov chain Monte Carlo settings
☒	☐	For hierarchical and complex designs, identification of the appropriate level for tests and full reporting of outcomes
☐	☒	Estimates of effect sizes (e.g. Cohen's d, Pearson's r), indicating how they were calculated

Our web collection on statistics for biologists contains articles on many of the points above.

Software and code

Policy information about availability of computer code

Data collection	Campbell Scientific LoggerNet 4
Data analysis	Microsoft Excel 2016, SAS JMP Pro 13 with Calculate Effect Sizes Add-in, custom image analysis software

For manuscripts utilizing custom algorithms or software that are central to the research but not yet described in published literature, software must be made available to editors and reviewers. We strongly encourage code deposition in a community repository (e.g. GitHub). See the Nature Research guidelines for submitting code & software for further information.

Data

Policy information about availability of data

All manuscripts must include a data availability statement. This statement should provide the following information, where applicable:

- Accession codes, unique identifiers, or web links for publicly available datasets
- A list of figures that have associated raw data
- A description of any restrictions on data availability

The data that support the findings of this study are available from the authors on reasonable request, see author contributions for specific data sets.

Field-specific reporting

Life sciences study design

All studies must disclose on these points even when the disclosure is negative.

Sample size	A sample size calculation was performed with assumptions of control group fresh mass of 35 g, experimental group fresh mass means of 40 g and standard deviations of 5 g, an enrollment ratio of 1, an alpha of 0.05, and a power of 80%; this calculation yielded a sample size of n=16 for each group; the experimental setup afforded n=12 for each group, so the experiment was repeated 3 times to achieve N=36 for each group. The statistical test was two-sided and requires noting in the manuscript as such along with inclusion of additional requested values for null hypothesis testing.
Data exclusions	Studentized residuals were calculated for the total data set of fresh mass, dry mass, and total leaf area, and data were excluded if they were >3 standard deviations away from the means. Data excluded from the statistical analyses are denoted in the raw data spreadsheets.
Replication	The experiment was repeated 3 times and produced successful replication of results for total leaf area in all 3 experiments. In repeated experiments 2 and 3, dry and fresh mass results were replicated with O-QD > R-QD > Control, whereas experiment 1 results showed R-QD > O-QD > Control. The cause of this reversal in fresh and dry mass trends between experiments 1 and 2 was not determined from the environmental data, but the integrated results for dry and fresh mass from all 3 experiments are consistent with the latter two experimental results.
Randomization	300 seeds were germinated, two per rockwool cube, among 150 rockwool cubes. 50 cubes were germinated in each of the three plant growth chambers, and the seedlings were thinned so that only one seedling remained per rockwool cube at 7 days after sowing (DAS). At this time, the 30 most uniform seedlings were selected from within each group of 50, and the remaining 20 were removed at that time. The experimental treatment continued thereafter for 21 DAS.
Blinding	Blinding was not possible because a single operator performed germination as well as data collection and analysis.

Reporting for specific materials, systems and methods

We require information from authors about some types of materials, experimental systems and methods used in many studies. Here, indicate whether each material, system or method listed is relevant to your study. If you are not sure if a list item applies to your research, read the appropriate section before selecting a response.

Materials & experimental systems

n/a	Involved in the study	
☒	☐	Antibodies
☒	☐	Eukaryotic cell lines
☒	☐	Palaeontology and archaeology
☐	☒	Animals and other organisms
☒	☐	Human research participants
☒	☐	Clinical data
☒	☐	Dual use research of concern

Methods

n/a	Involved in the study	
☒	☐	ChIP-seq
☒	☐	Flow cytometry
☒	☐	MRI-based neuroimaging

Animals and other organisms

Policy information about studies involving animals; ARRIVE guidelines recommended for reporting animal research

Laboratory animals	*For laboratory animals, report species, strain, sex and age OR state that the study did not involve laboratory animals.*
Wild animals	*Provide details on animals observed in or captured in the field; report species, sex and age where possible. Describe how animals were caught and transported and what happened to captive animals after the study (if killed, explain why and describe method; if released, say where and when) OR state that the study did not involve wild animals.*
Field-collected samples	*For laboratory work with field-collected samples, describe all relevant parameters such as housing, maintenance, temperature, photoperiod and end-of-experiment protocol OR state that the study did not involve samples collected from the field.*
Ethics oversight	No ethical approval or guidance was required because this study involved only plants.

Note that full information on the approval of the study protocol must also be provided in the manuscript.
